# Arthroscopic Medial Meniscus Posterior Root Repair with Centralization Using Knotless Suture Anchors

**DOI:** 10.1016/j.eats.2021.12.019

**Published:** 2022-03-28

**Authors:** Thomas Y. Wu

**Affiliations:** Ventura County Medical Center, Ventura, California, U.S.A.

## Abstract

Medial meniscus posterior root tears can lead to rapid progression of knee arthritis because of loss of the stress distribution function of the meniscus. Medial meniscus root repair can restore stress distribution and improve clinical outcome. In cases of medial meniscus root tears with meniscal extrusion, centralization may help reduce extrusion and protect the root repair. Presented here is a technique for transtibial medial meniscus root repair with centralization using knotless suture anchors, building on previously developed techniques.

Medial meniscus posterior root tears can lead to rapid progression of knee arthritis.^1^ This is likely due to loss of stress distribution via the hoop mechanism, leading to a biomechanical state similar to total meniscectomy.^2^ Medial meniscus root repair can restore stress distribution provided by the medial meniscus and improve clinical outcome.^2^, ^3^, ^4^, ^5^, ^6^ Medial meniscus root tears are associated with increased extrusion over time.^7^ Meniscal extrusion is an independent risk factor for the progression of osteoarthritis.^8^ In cases of medial meniscus root tears with meniscal extrusion, centralization may help reduce extrusion and protect the root repair.^3^ Thus medial meniscus root repair with centralization is indicated for medial meniscus posterior root tears with extrusion and without significant arthritis (Kellgren-Lawrence grade 0-2),^9^ which is often seen with nontraumatic or minimally-traumatic tears in older patients, typically over the age of 40. Presented here is a technique for transtibial medial meniscus root repair with centralization using knotless suture anchors, building on previously developed techniques.^3^^,^^10^, ^11^, ^12^

## Surgical Technique

**Fig 1:**
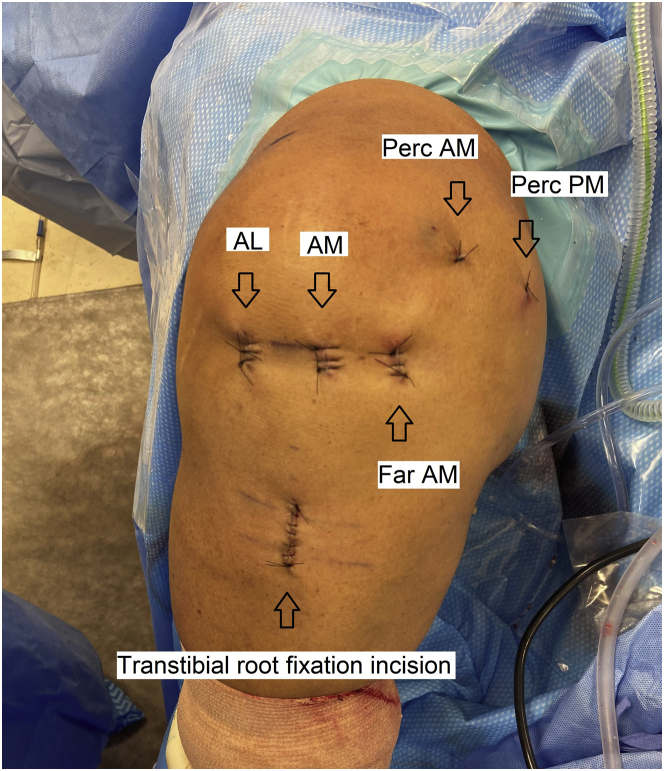
Incisions used for the procedure (right knee, supine position). The AL and AM portals are adjacent to the patellar tendon. AL, anterolateral portal; AM, anteromedial portal; Far AM, far anteromedial portal; Perc AM, percutaneous anteromedial portal for insertion and tensioning of anteromedial centralization anchor; Perc PM, percutaneous posteromedial portal for insertion and tensioning of posteromedial centralization anchor.

Arthroscopy is performed with the patient in the supine position (Fig 1). Anterolateral and anteromedial portals are established adjacent to the patellar tendon. A far anteromedial portal is established for additional access.

**Fig 2:**
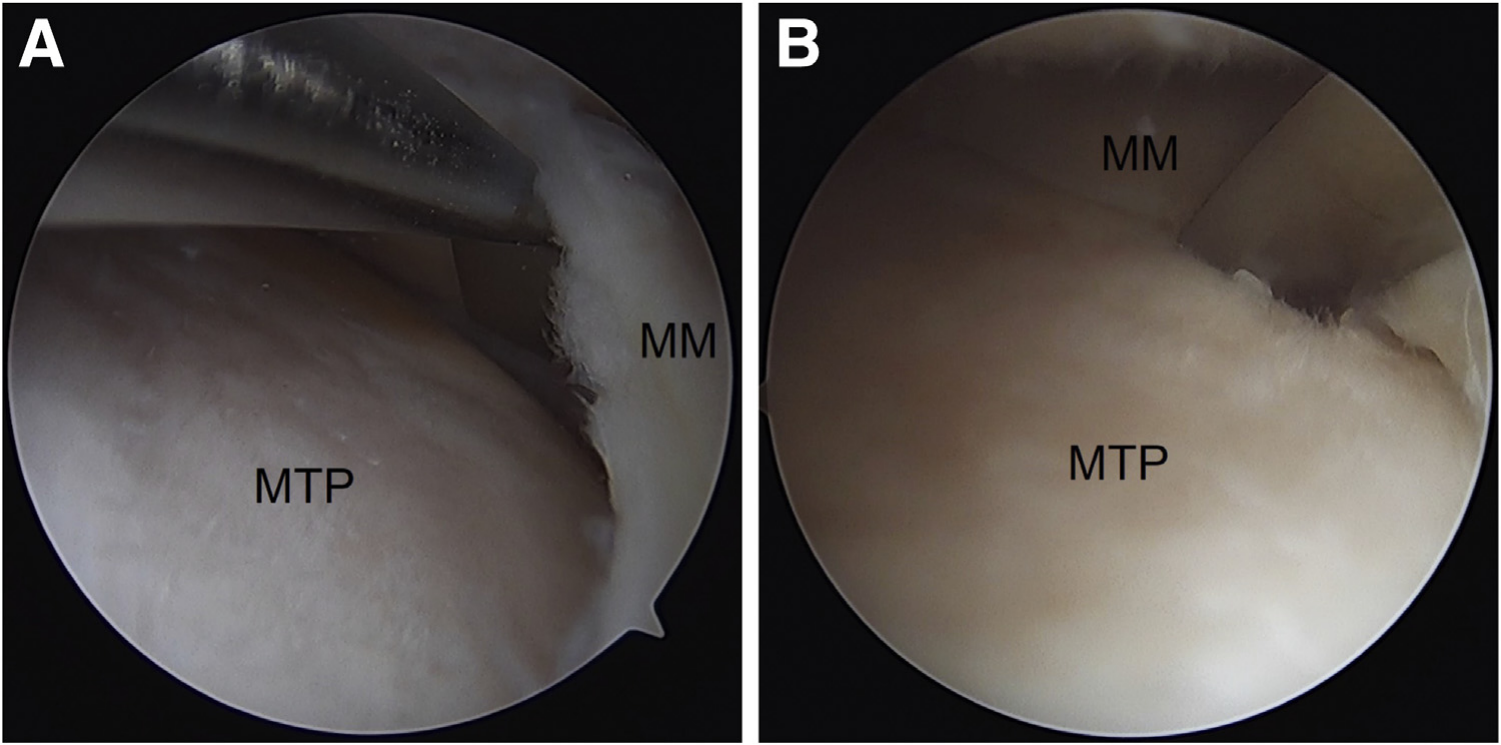
A Bankart elevator is used to mobilize the meniscus along the tibial rim (right knee). (A) Bankart elevator via the AL portal (viewing from AM portal). (B) Bankart elevator via far AM portal (viewing from AL portal). AL, anterolateral; AM, anteromedial; MM, medial meniscus; MTP, medial tibial plateau.

The medial meniscus is mobilized using a Bankart elevator along the tibial rim. This can be done via the far anteromedial portal and the anterolateral portal (Fig 2).

**Fig 3:**
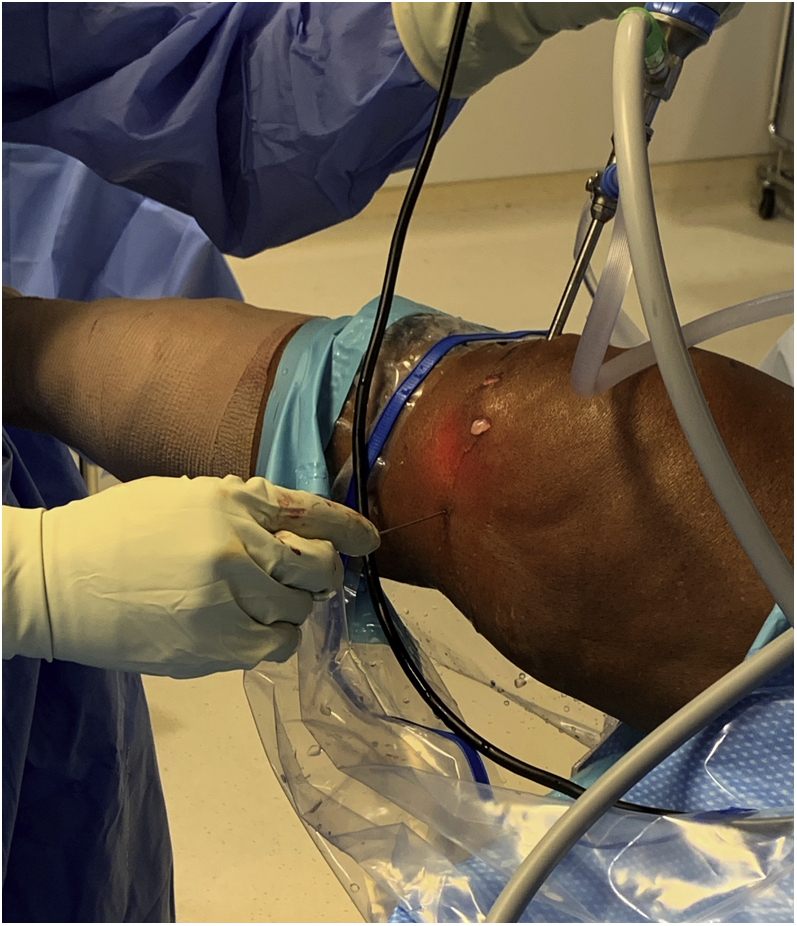
An 18-gauge spinal needle can be used to percutaneously partially release the MCL while placing a valgus stress to increase the working space by a couple of millimeters (right knee, supine position). MCL, medial collateral ligament.

If the working space remains tight, an 18-gauge spinal needle is used to percutaneously partially release the medial collateral ligament (MCL) to increase the working space by a couple of millimeters. This is done at the joint line with knee in near extension and with a valgus stress (Fig 3). This step can also be done before mobilizing the meniscus if there is insufficient space, but be aware that valgus laxity can increase further after meniscus mobilization from the tibial rim.

**Fig 4:**
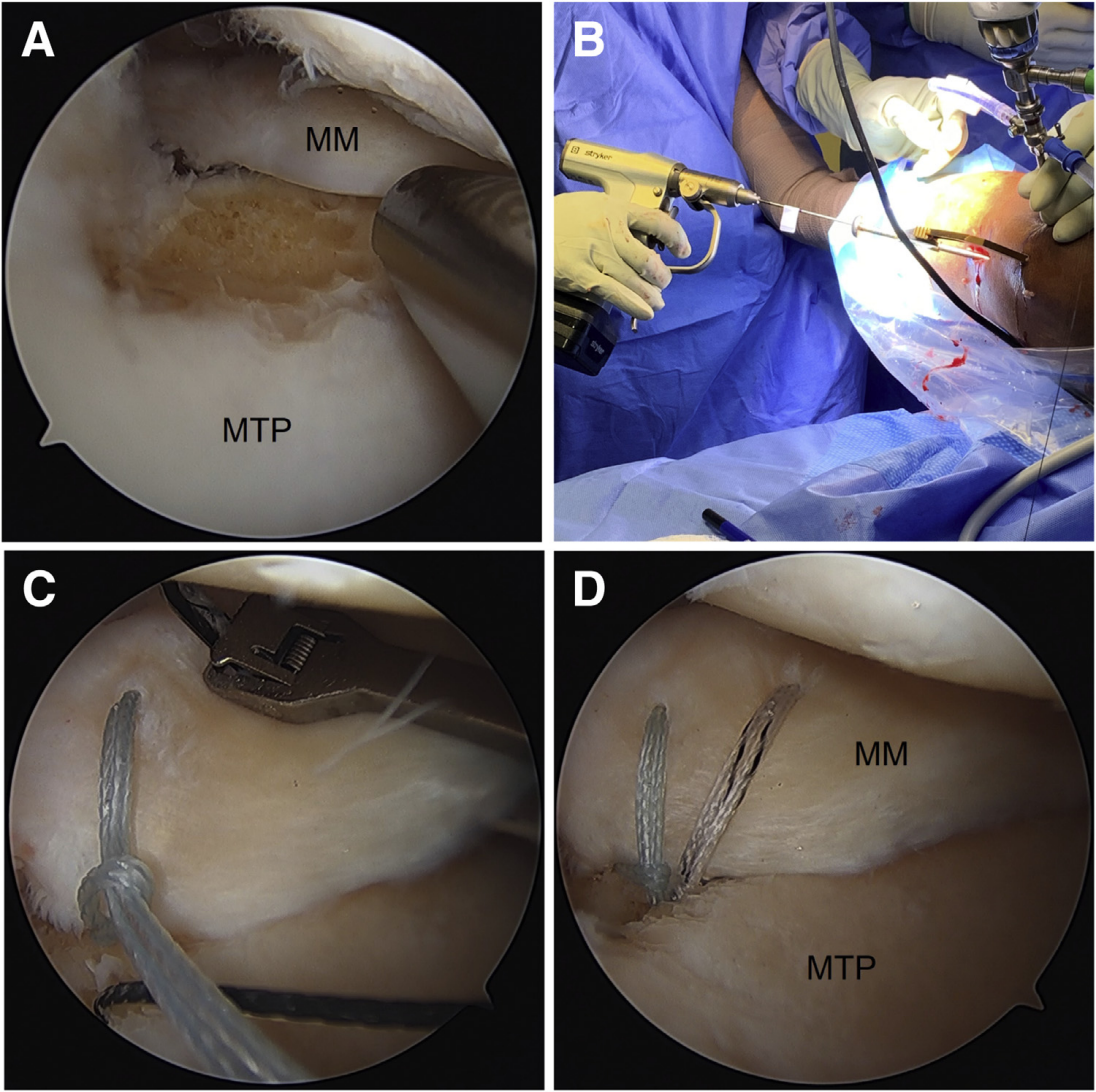
Right knee. (A) A 4.5 mm angled shaver is used to expose bone for healing under the meniscal root (viewing from AL portal). (B) Using a standard tip-to-tip ACL tibial guide, the tibial tunnel for the root repair is drilled using a 2.4mm guide pin. Note that a white sticker was placed on the guide pin at a location that would indicate that the pin has been inserted the full depth of the tunnel once the sticker reaches the bullet of the guide. (C) Fixation sutures are placed in the meniscal root in cinch fashion via the anteromedial portal (viewing from AL portal). (D) The tails of the root fixation sutures are shuttled down the tibial tunnel. ACL, anterior cruciate ligament; AL, anterolateral; MM, medial meniscus; MTP, medial tibial plateau.

The meniscus is grasped to assess excursion to help determine the location of the tibial tunnel for the root repair. A 4.5 mm angled shaver (Stryker, Kalamazoo, MI) is used to expose bone for healing under the meniscal root (Fig 4A). The shaver is run on the underside of the meniscal root to stimulate healing.

Using a standard tip-to-tip anterior cruciate ligament (ACL) tibial guide (Acufex Director; Smith & Nephew, Watford, Hertfordshire, UK) placed through the anteromedial portal, the tibial tunnel for the root repair is drilled using a 2.4 mm standard guide pin through an incision over the anteromedial proximal tibia (Fig 4B). Care is taken to avoid injury to the posterior neurovascular structures because of overpenetration. The guide pin is removed, and a straight SutureLasso (Arthrex, Naples, FL) is placed through the bullet of the guide to pass a nitinol wire loop up the tunnel. The loop end is pulled out through the far anteromedial portal. Alternatively, a retractable suture passer can be used to bring a shuttling suture up through the tunnel and out through the far anteromedial portal.

Next, fixation sutures are placed in the meniscal root. While visualizing from the anterolateral portal, a no. 0 FiberLink (Arthrex) and a no. 0 TigerLink (Arthrex) are placed using a Knee Scorpion (Arthrex) in cinch fashion via the anteromedial portal (Fig 4C). The tails of the sutures are shuttled down the tibial tunnel (Fig 4D).

**Fig 5:**
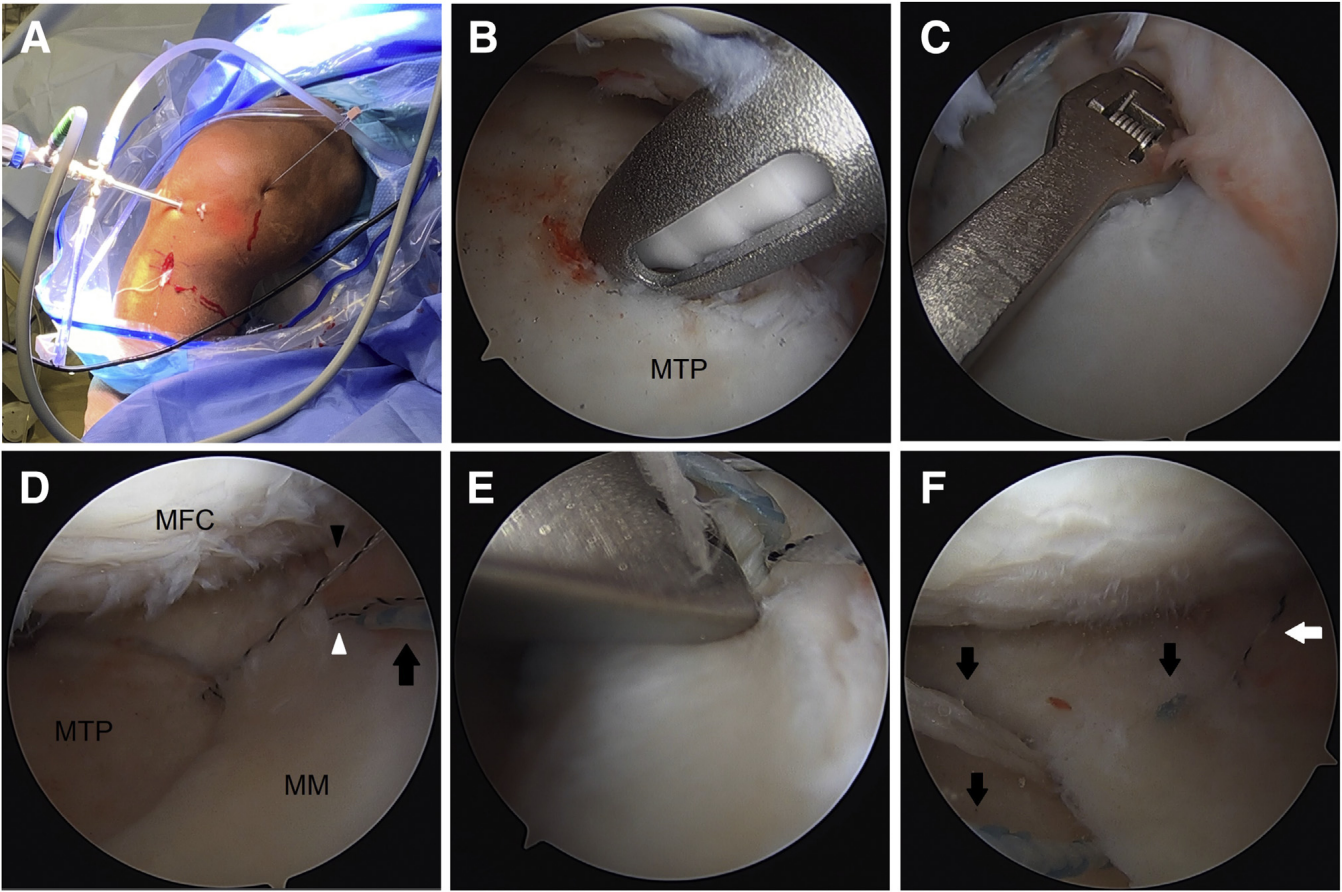
Right knee. (A) Needle localization for percutaneous posteromedial portal for the posteromedial centralization anchor. (B) A 3mm Knotless BioComposite SutureTak anchor is placed about 3 mm in from the posteromedial tibial rim (viewing from AM portal). (C) A 2-0 FiberWire shuttling suture is placed through the meniscocapsular junction just anterior to the anchor site using the Knee Scorpion while the meniscal root is held reduced by pulling traction on the root sutures (viewing from AM portal). (D) The fixation suture of the anchor (*black arrow*) and the leading limb of the anchor’s shuttling suture (*white arrowhead*), which is in tape form, are shuttled through the meniscocapsular junction using the 2-0 FiberWire (viewing from AM portal). *Black arrowhead* is the trailing limb of the anchor’s shuttling suture. (E) The fixation suture is passed back down through the meniscus 3 to 4 mm from the meniscocapsular junction and a little anterior to the anchor site using the Knee Scorpion oriented upside-down (viewing from AM portal). (F) The anchor’s shuttling suture (*white arrow*) is used to shuttle the fixation suture (*black arrows*) through the anchor, then up through the meniscocapsular junction, and out the percutaneous portal without final tensioning (viewing from AM portal). AM, anteromedial; MFC, medial femoral condyle; MM, medial meniscus; MTP, medial tibial plateau.

Next, the posteromedial centralization anchor is placed. The arthroscope is moved to the anteromedial portal. It may be necessary to debride some anteromedial synovium for visualization. With the knee in flexion, the long needle from the percutaneous SutureTak kit (Arthrex) is used to localize the percutaneous incision for placement of the anchor. The needle is placed posterior to the MCL (Fig 5A). Initial localization may be done more easily using an 18-gauge spinal needle before placing the long needle from the percutaneous kit. The nitinol wire is placed through the needle. Skin incision is made, and the dilator is placed over the nitinol wire. The drill guide is placed over the dilator. A probe is placed through the far anteromedial portal to retract the medial meniscus to visualize the posteromedial tibial plateau rim. The hole for the anchor is drilled about 3 mm in from the posteromedial rim, and a 3 mm Knotless BioComposite SutureTak anchor (Arthrex) is placed (Fig 5B).

Through a PassPort cannula (Arthrex) in the anterolateral portal, a 2-0 FiberWire (Arthrex) shuttling suture is placed through the meniscocapsular junction just anterior to the anchor site using the Knee Scorpion while the meniscal root is held reduced by pulling traction on the root sutures (Fig 5C). The fixation suture of the anchor and the leading limb of the anchor’s shuttling suture, which is in tape form, are shuttled through the meniscocapsular junction using the 2-0 FiberWire by first tying the 2-0 FiberWire around them (Fig 5D).

Next, the fixation suture is passed back down through the meniscus 3 to 4 mm from the meniscocapsular junction and a little anterior to the anchor site using the Knee Scorpion oriented upside-down (Fig 5E). Alternatively, a 2-0 FiberWire can be passed in the normal orientation and then used to shuttle the fixation suture.

The looped trailing end of the anchor’s shuttling suture is then used to shuttle a 2-0 FiberWire shuttling suture in through the percutaneous portal and out the anterolateral portal. The 2-0 FiberWire is moved from the anterolateral portal to the far anteromedial portal and used to shuttle the leading end of the anchor’s shuttling suture back out the percutaneous portal so that later shuttling of the fixation suture through the anchor and tensioning can be done in an optimal direction of pull for the knotless anchor. This eases shuttling and tensioning and helps avoid failure of the anchor during those steps. The anchor’s shuttling suture is then used to shuttle the fixation suture through the anchor, then up through the meniscocapsular junction, and out the percutaneous portal without final tensioning (Fig 5F).

**Fig 6:**
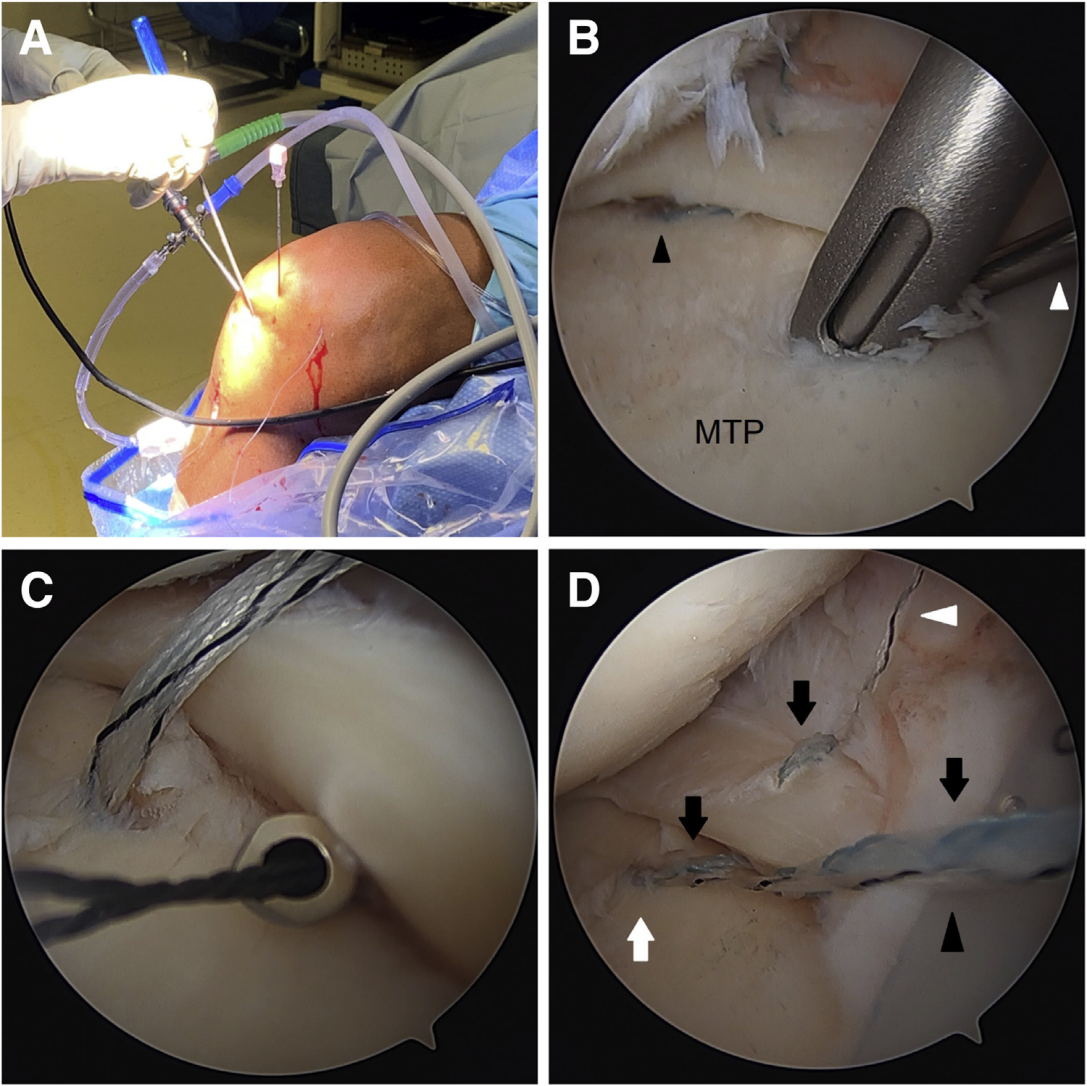
Right knee. (A) Needle localization for percutaneous anteromedial portal for the anteromedial centralization anchor. (B) A 3mm Knotless BioComposite SutureTak anchor is placed about 3 mm in from the anteromedial tibial rim (viewing from AL portal). *Black arrowhead* is the posteromedial centralization anchor. *White arrowhead* is a probe retracting the medial meniscus. (C) With the meniscal root held reduced by pulling traction on the root sutures, a 25° QuickPass Lasso is used via the far AM portal to pass a 2-0 FiberWire shuttling suture at the meniscocapsular junction just anterior to the anchor site (viewing from AL portal). (D) Shuttling is done so that the fixation suture is passed up through the meniscocapsular junction, down through the meniscus 3 to 4 mm from the meniscocapsular junction and a little anterior to the anchor, through the anchor, and then up through the meniscocapsular junction, and out the percutaneous portal without final tensioning (viewing from AL portal). *Black arrows* are the fixation suture. *White arrowhead* is the leading limb of the anchor’s shuttling suture, which is in tape form. *Black arrowhead* is the trailing limb of the anchor’s shuttling suture. *White arrow* is the anteromedial centralization anchor. AL, anterolateral; AM, anteromedial; MTP, medial tibial plateau.

The same procedure is now repeated for the anteromedial centralization anchor. The arthroscope is placed back in the anterolateral portal. The anchor is placed percutaneously 3mm in from the anteromedial rim of the tibial plateau (Figs 6 A and B). The PassPort cannula is placed in the anteromedial portal. With the meniscal root held reduced by pulling traction on the root sutures, a 25-degree QuickPass Lasso (Arthrex) is used via the far anteromedial portal to pass the fixation suture and the leading limb of the anchor’s shuttling suture through the meniscocapsular junction just anterior to the anchor site (Fig 6C). The Lasso is then used to pass a 2-0 FiberWire shuttling suture 3 to 4 mm from the meniscocapsular junction and a little anterior to the anchor. The shuttling suture is used to pass the fixation suture down through the meniscus.

The trailing end of the anchor’s shuttling suture is then used to shuttle a 2-0 FiberWire shuttling suture in through the percutaneous portal, which is then used to shuttle the leading end of the anchor’s shuttling suture back out the percutaneous portal. The anchor’s shuttling suture is then used to shuttle the fixation suture through the anchor, up through the meniscocapsular junction, and out the percutaneous portal without final tensioning (Fig 6D).

**Fig 7:**
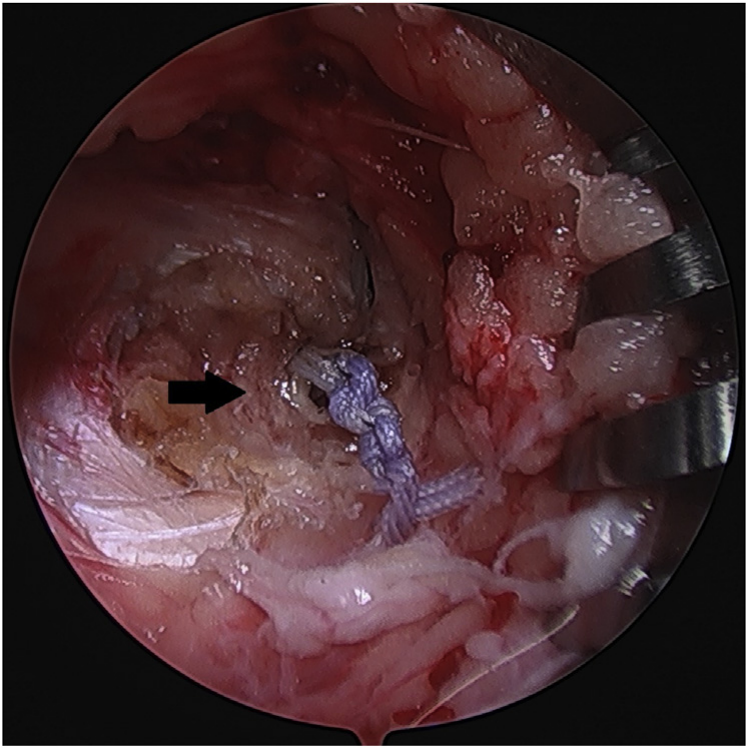
The posterior root sutures are fixated using a 4.75 mm PEEK SwiveLock anchor (*arrow*) on the anteromedial proximal tibia about 1 cm distal to the suture tunnel while ensuring that the medial compartment is not loaded, right knee. The central stay suture of the SwiveLock is tied to the root sutures to prevent slippage. PEEK, polyether ether ketone.

The posterior root sutures are then fixated using a 4.75 mm PEEK (polyether ether ketone) SwiveLock anchor (Arthrex) on the anteromedial proximal tibia about 1 cm distal to the suture tunnel after drilling and tapping. While ensuring that the medial compartment is not loaded, the root sutures are individually tensioned before fixation. The central stay suture of the SwiveLock is tied to the root sutures to prevent slippage (Fig 7).

**Fig 8:**
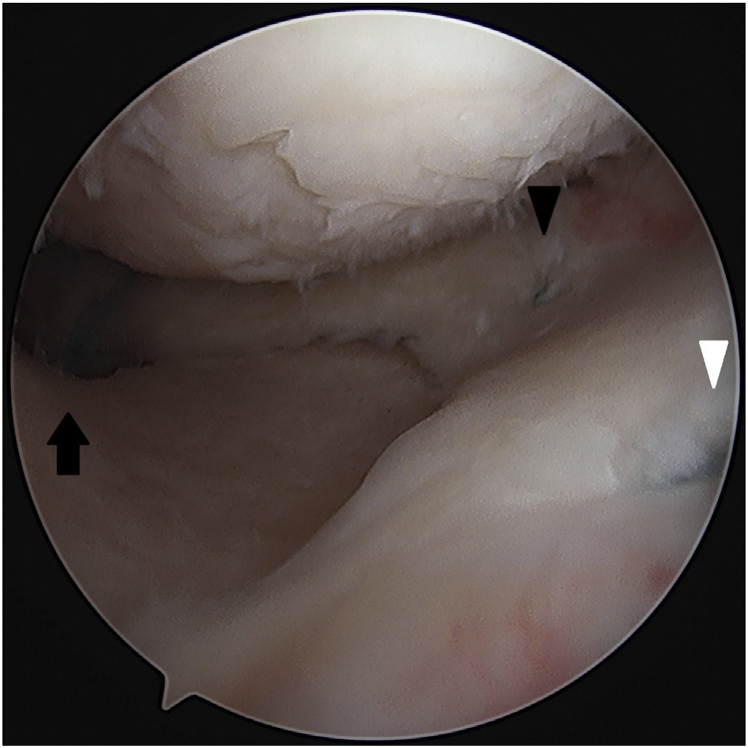
Final construct, right knee, viewing from AL portal. *Black arrow* is the medial meniscus posterior root repair. *Black arrowhead* is posteromedial centralization. *White arrowhead* is anteromedial centralization. AL, anterolateral.

**Fig 9:**
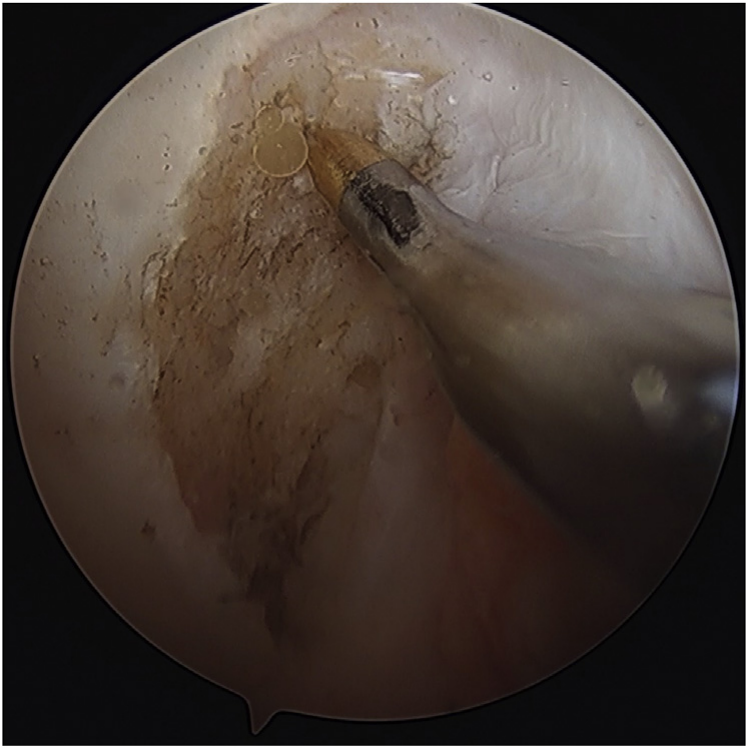
Microfracture of the lateral intercondylar notch is performed to help facilitate meniscal healing, right knee, viewing from AM portal. AM, anteromedial.

Final tensioning of the centralization sutures is done, and the sutures are cut flush to the meniscus (Fig 8). Microfracture of the lateral intercondylar notch is then performed to help facilitate meniscal healing (Fig 9).

The surgical technique is shown in Video 1. Indications and key steps of the procedure are summarized in Table 1. Advantages and limitations of the technique are summarized in Table 2.

**Table 1:** Indication and Key Steps

Indication
Medial meniscus posterior root tear with extrusion, without significant arthritis (Kellgren-Lawrence 0-2).
Key steps
1. Create anteromedial and anterolateral portals adjacent to patellar tendon, create far anteromedial portal.
2. Mobilize medial meniscus using a Bankart elevator.
3. If needed, percutaneously partially release MCL to improve working space.
4. Expose bone at root repair site using 4.5 mm angled shaver.
5. Drill 2.4 mm tibial tunnel for root repair using standard tip-to-tip ACL guide.
6. Pass root fixation sutures through meniscus in cinch fashion and shuttle down the tibial tunnel.
7. Percutaneously place knotless posteromedial centralization anchor.
8. Using shuttling technique described, pass the anchor's fixation suture up through meniscocapsular junction, down through meniscus, in through anchor, up through meniscocapsular junction, and out through original percutaneous portal.
9. Repeat similar procedure for anteromedial centralization anchor.
10. Fixate the posterior root sutures, ensuring that the medial compartment is not loaded.
11. Final tension the sutures of the centralization anchors.
12. Microfracture the lateral intercondylar notch to facilitate healing.

ACL, anterior cruciate ligament; MCL, medial collateral ligament.

**Table 2:** Advantages and Limitations of the Technique

Advantages
Use of anchors for centralization avoids potential tunnel convergence and fixation crowding and reduces movement at fixation spots compared to transtibial far cortical fixation.
Use of knotless anchors avoids abrasion of articular surface due to knots.
Use of the Knotless SutureTak provides more reliable deployment compared to all-suture knotless anchors.
Biocomposite anchor for centralization avoids long-term abnormal limitation to meniscus motion and is likely less damaging to the joint in case of pull-out compared to PEEK or metallic implants.
Points of fixation for centralization are independent from each other.
Percutaneous placement of centralization anchors allows optimal trajectories.
The shuttling technique described allows optimal direction of pull for shuttling through the anchors and tensioning of the anchors' fixation sutures. This eases shuttling and tensioning and helps avoid anchor failure during those steps.
Non-bulky ACL guide for root repair allows freedom for precise tunnel placement.
Bone preparation using angled shaver is simple and avoids creation of nonanatomic oblique socket.
PEEK SwiveLock fixation for meniscal root provides good tensioning control and long-term fixation that serves as backup for biologic healing
Limitations
In case of implant pullout, biocomposite centralization anchors may be more damaging to the joint than all-suture anchors.
Suture cutout can cause iatrogenic damage to the meniscus.
Neurovascular injury risks are present for tibial tunnel drilling and portal creation.

ACL, anterior cruciate ligament; PEEK, polyether ether ketone.

## Postoperative Rehabilitation

Patients are kept non-weightbearing on the operative extremity in an extension brace for 6 weeks, although they are instructed to bear weight at any point if needed to avoid falls. Mobilization of the knee is limited to 0° to 90° of flexion for 6 weeks. After 6 weeks, weightbearing and range of motion are advanced as tolerated, and the brace is opened to allow motion and then weaned. Squatting with knees flexed more than 90°, as well as running are avoided for 4 months.

## Discussion

The advantages of this technique vary depending on what it is being compared to.^3^^,^^10^, ^11^, ^12^ Regarding the centralization portion of the procedure, the advantages of using a knotless biocomposite anchor are as follows. When compared to transtibial far cortical fixation, use of anchors avoids potential problems with convergence with other tunnels created during surgery. It avoids crowding of fixation on the anterior cortex of the proximal tibia. There is likely less movement at the fixation spots because of minimal distance between the meniscal tissue being fixated and the anchor point. When compared to fixation using anchors that require knot-tying, knotless anchors avoid abrasion of the articular surface caused by knots. When compared to all-suture knotless anchors, which must properly expand during deployment to achieve fixation such as the Knotless FiberTak (Arthex), the Knotless Biocomposite SutureTak is more reliable during surgery. The biocomposite version of the knotless SutureTak is chosen because it avoids long-term abnormal limitation to meniscus motion, and in the event of fixation failure, the implant is likely less damaging in the joint compared to PEEK or metallic implants.

The specific method in which the knotless anchor is used in this technique has advantages. When compared to a technique that uses multiple knotless anchors in which the fixation suture from 1 anchor is shuttled through another anchor, this technique keeps each fixation point independent so that if there is loosening at one fixation point, other fixation points are not directly affected. The use of percutaneous insertion portals for the centralization anchors allows optimal trajectories for placement of the anchors that is much less constrained compared to techniques that rely on anchor placement via a standard anteromedial or far anteromedial portal. Also, using the shuttling technique described here for routing the leading limb of the anchor’s shuttling suture back through the percutaneous portal through which the anchor was placed, one can shuttle and tension the fixation suture in the most optimal direction for the anchor. This eases shuttling and tensioning and helps avoid failure of the anchor during those steps.

Regarding the root repair portion of the procedure, use of a non-bulky, tip-to-tip ACL tibial drill-guide allows precise placement of the tibial tunnel based on anatomy and meniscal mobility without the constraint imposed by some bulkier meniscal root guides. Bone preparation using an angled shaver is simple and avoids creation of a necessarily oblique and nonanatomic socket when using the FlipCutter (Arthrex) technique.^11^

The PEEK SwiveLock is chosen for fixation of the meniscal root because, compared to the biocomposite version, it is expected to provide longer-term fixation, which can serve as a back-up to the healed meniscal root, which may not be as strong as a normal root attachment. Tying of the root sutures to the central stay suture of the SwiveLock helps prevent slippage of the interference-type fixation. Compared to knot-tying over a cortical button, this fixation method provides greater tensioning control.

Risks of this technique include pull-out of centralization anchors, fixation suture cut-out through meniscal tissue, saphenous nerve injury during percutaneous portal creation, and posterior neurovascular injury during drilling of the tibial tunnel for the root repair.

Although it makes logical sense that centralization would help reduce extrusion and protect medial meniscus posterior root repairs, thus reducing failure rates and arthritis progression, studies will be needed to confirm good outcomes with this procedure. Currently, because of it being a relatively new procedure, very limited evidence exists showing that it improves clinical outcomes.^13^ Furthermore, comparative studies, ideally randomized controlled, would be needed to demonstrate superiority of root repair with centralization over root repair alone.

## Supplementary Data

ICMJE author disclosure forms

Video 1 Arthroscopic medial meniscus posterior root repair with centralization using knotless suture anchors is indicated in root tears with extrusion and without significant arthritis. This is a right knee. The patient is in the supine position. Portals used are anterolateral and anteromedial portals adjacent to the patellar tendon, a far anteromedial portal, and percutaneous posteromedial and anteromedial portals for insertion of centralization anchors. The medial meniscus is mobilized using a Bankart elevator along the tibial rim. If the working space remains tight, an 18-gauge spinal needle is used to percutaneously partially release the MCL. A 4.5 mm angled shaver is used to expose bone for healing. The shaver is run on the underside of the meniscus to stimulate healing. Using a standard tip-to-tip ACL guide, the tibial tunnel for the root repair is drilled using a 2.4 mm guide pin. A straight SutureLasso is placed through the bullet of the guide to pass a nitinol wire shuttling loop. A no. 0 FiberLink and a no. 0 TigerLink are placed through the meniscal root using a Knee Scorpion in cinch fashion. The tails of the sutures are shuttled down the tibial tunnel. Next, the posteromedial centralization anchor is placed. The long needle from the percutaneous SutureTak kit is used to localize the incision. Over a nitinol wire, the needle is switched out for the drill guide. A 3 mm Knotless BioComposite SutureTak anchor is placed about 3 mm in from the posteromedial tibial rim. A 2-0 FiberWire shuttling suture is placed through the meniscocapsular junction just anterior to the anchor site using the Knee Scorpion while the meniscal root is held reduced by pulling traction on the root sutures. The fixation suture of the anchor and the leading limb of the anchor’s shuttling suture are shuttled through the meniscocapsular junction. The fixation suture is passed back down through the meniscus using the Knee Scorpion oriented upside-down. Alternatively, a 2-0 FiberWire can be passed in the normal orientation and used to shuttle the fixation suture. The looped trailing end of the anchor’s shuttling suture is used to shuttle a 2-0 FiberWire shuttling suture in through the percutaneous portal and out the anterolateral portal. The 2-0 FiberWire is moved from the anterolateral portal to the far anteromedial portal and used to shuttle the leading end of the anchor’s shuttling suture back out the percutaneous portal so that it will have an optimal direction of pull. The anchor’s shuttling suture is then used to shuttle the fixation suture through the anchor, up through the meniscocapsular junction, and out the percutaneous portal. The same procedure is now repeated for the anteromedial centralization anchor. The anchor is placed percutaneously 3 mm in from the anteromedial tibial rim. Suture passage through the meniscus is performed using a 25° QuickPass Lasso. The same shuttling sequence is done. The posterior root sutures are then fixated using a 4.75 mm PEEK SwiveLock on the anteromedial proximal tibia about 1 cm distal to the suture tunnel, while ensuring that the medial compartment is not loaded. The central stay suture of the SwiveLock is tied to the root sutures to prevent slippage. Final tensioning of the centralization sutures is done. Microfracture of the lateral intercondylar notch is performed to help facilitate meniscal healing. MCL, medial collateral ligament; ACL, anterior cruciate ligament; PEEK, polyether ether ketone.
